# Retraction Note: *Tridax procumbens* flavonoids: a prospective bioactive compound increased osteoblast differentiation and trabecular bone formation

**DOI:** 10.1186/s40659-023-00450-5

**Published:** 2023-07-07

**Authors:** Md. Abdullah Al Mamun, Mohammad Jakir Hosen, Amina Khatun, M. Masihul Alam, Md. Abdul Alim Al‑Bari

**Affiliations:** 1 Department of Genetic Engineering and Biotechnology, Shahjalal University of Science and Technology, Sylhet, 3114 Bangladesh; 2 Department of Anthropology, Shahjalal University of Science and Technology, Sylhet, 3114 Bangladesh; 3 Department of Applied Nutrition and Food Technology, Islami University, Kustia, 7003 Bangladesh; 4 Department of Pharmacy, Rajshahi University, Rajshahi, 6205 Bangladesh

**Retraction** **:** **Biol Res (2017) 50:28** **https://doi.org/10.1186/s40659-017-0134-7**

The Editor in Chief has retracted this article because of significant text overlap with a previous work by the same authors [1]. Further investigation by the publisher found that images in Figure 5 appear to be all derived from the same picture with some small differences in brightness, field of view or magnification, but are described as representing different conditions. The authors were unable to explain this overlap or provide evidence of ethics approval for the animal experiments from Ethical and Animal Care and Use Committee of Shahjalal University of Science and Technology, Sylhet as stated in the article.

Therefore, the Editor has lost confidence in the data presented in this article.

Author Md. Abdullah Al Mamun does not agree to this retraction. Authors Mohammad Jakir Hosen, Amina Khatun, M. Masihul Alam and Md. Abdul Alim Al-Bari have not responded to correspondence from the Editor or Publisher about this retraction.
